# Task-Related Hemodynamic Response Alterations During Slacklining: An fNIRS Study in Advanced Slackliners

**DOI:** 10.3389/fnrgo.2021.644490

**Published:** 2021-03-03

**Authors:** Oliver Seidel-Marzi, Susanne Hähner, Patrick Ragert, Daniel Carius

**Affiliations:** ^1^Institute for General Kinesiology and Exercise Science, Faculty of Sport Science, University of Leipzig, Leipzig, Germany; ^2^Department of Neurology, Max Planck Institute for Human Cognitive and Brain Sciences, Leipzig, Germany

**Keywords:** hemodynamic response, functional near-infrared spectroscopy, slacklining, complex movement, experienced athletes

## Abstract

The ability to maintain balance is based on various processes of motor control in complex neural networks of subcortical and cortical brain structures. However, knowledge on brain processing during the execution of whole-body balance tasks is still limited. In the present study, we investigated brain activity during slacklining, a task with a high demand on balance capabilities, which is frequently used as supplementary training in various sports disciplines as well as for lower extremity prevention and rehabilitation purposes in clinical settings. We assessed hemodynamic response alterations in sensorimotor brain areas using functional near-infrared spectroscopy (fNIRS) during standing (ST) and walking (WA) on a slackline in 16 advanced slackliners. We expected to observe task-related differences between both conditions as well as associations between cortical activity and slacklining experience. While our results revealed hemodynamic response alterations in sensorimotor brain regions such as primary motor cortex (M1), premotor cortex (PMC), and supplementary motor cortex (SMA) during both conditions, we did not observe differential effects between ST and WA nor associations between cortical activity and slacklining experience. In summary, these findings provide novel insights into brain processing during a whole-body balance task and its relation to balance expertise. As maintaining balance is considered an important prerequisite in daily life and crucial in the context of prevention and rehabilitation, future studies should extend these findings by quantifying brain processing during task execution on a whole-brain level.

## Introduction

Maintaining balance is considered a basic requirement for numerous activities of daily life over the lifespan, e.g., to reduce the rate of falls among elderly people (Clemson et al., 2012). According to current literature, balance ability is based on various processes of motor control in complex neural networks of subcortical and cortical brain structures (Surgent et al., 2019). On the one hand, it has been shown on a structural level that gray matter (GM) density predicts balance stability irrespective of age (Boisgontier et al., 2016). Furthermore, balance training is associated with neuroplastic adaptations in motor-related cortical structures not only in GM but also in white matter (WM) (Taubert et al., 2010, 2016). For example, Taubert et al. (2010) revealed microstructural cortical changes in GM and WM after practicing a complex balance task over a period of several weeks. Furthermore, using the same complex balance task, Taubert et al. (2016) found that local motor cortical thickness in primary motor cortex (M1) increased even after 1 h of practice, indicating neuroplastic adaptations on a relatively short time scale. On the other hand, balance training is also capable of inducing functional adaptations as previously reported for alterations in brain activity in M1 (Ruffieux et al., 2018) and in resting-state functional connectivity between striatum and other brain areas (Magon et al., 2016).

These findings indicate that not only brain function but also brain morphology plays a crucial role for the motor control of maintaining balance. Previous knowledge on these processes has mainly resulted from investigations using established brain imaging methods such as magnetic resonance imaging (MRI) (Taubert et al., 2010, 2016; Magon et al., 2016; Ruffieux et al., 2018). However, in order to gain a better and more comprehensive understanding about neural correlates of whole-body balance tasks, it is essential to capture functional brain adaptations even while performing balance tasks. One conceivable methodological option to approach this goal is the use of wearable non-invasive brain imaging methods such as electroencephalography (EEG) and functional near-infrared spectroscopy (fNIRS).

Although EEG measurements can be limited by a low spatial resolution and artifact contamination, this technique has distinct advantages in temporal resolution as compared to MRI (Seidel-Marzi and Ragert, 2020). Therefore, numerous previous studies used EEG with source localization in order to detect activation and connectivity between cortical regions during balancing tasks (see Wittenberg et al., 2017 for a review). For example, Hülsdünker et al. (2015b) investigated cortical theta activity during nine balance tasks with varying difficulties. The authors found that theta power increased in frontal, central, and parietal regions of the cortex as balance tasks became more demanding. In a subsequent study, the same balance tasks were performed with eyes closed to investigate the individual alpha peak frequency (iAPF) as well as power in theta, lower alpha, and upper alpha frequency bands (Hülsdünker et al., 2015a). Here, the results revealed a global increase in iAPF, a decrease in lower alpha power as well as an increase in midline theta power with higher demands on balance control. While these studies contributed valuable insights in the involvement of the cerebral cortex in balance control by identifying specific cortical regions, another recent study focused on the interactions within and between relevant cortical regions (Mierau et al., 2017). In this study, findings indicate that at least two functional cortical networks contribute to an optimization of balance control (Mierau et al., 2017). According to the authors, the theta network facilitates the processing of somatosensory information as well as the planning and execution of motor responses, whereas the alpha network is assumed to support visual information processing required for optimal body stability.

Beyond the application of EEG in balance research, fNIRS has also been frequently used and has therefore been established as a promising technique in this context. This optical imaging method has distinct advantages over conventional brain imaging techniques such as EEG and MRI with regards to complex motor tasks and thus, has already been applied in a number of studies during the execution of whole-body motor tasks (see Seidel-Marzi and Ragert, 2020 for a review). In a recent systematic review, Herold et al. (2017b) summarized previous studies using fNIRS during balance tasks. The authors suggest that challenging balance tasks are primarily related to an increased activity in motor-related brain areas such as prefrontal cortex (PFC) and supplementary motor area (SMA). In another recent study, Seidel et al. (2017) investigated learning-induced changes in brain activity by means of fNIRS during a complex balance task. The authors found a learning-induced decreased brain activity in M1 and inferior parietal lobe (IPL), and, moreover, learning rates were correlated with fNIRS changes in these brain regions.

Previous knowledge on balance-related brain activity and structural changes resulted from studies using specific instruments for research purposes such as balance boards or tilt platforms, which are capable of investigating static balance. A further option for balance assessment and training is slacklining, which requires dynamic balance abilities. Slacklining is frequently used as supplementary training in various sports disciplines (Granacher et al., 2010; Santos et al., 2016; Trecroci et al., 2018) as well as for lower extremity prevention and rehabilitation purposes in clinical settings (Gabel and Mendoza, 2013; Gabel et al., 2015). Moreover, slacklining itself is core of several sports disciplines such as longlining and tricklining. Indeed, based on several previous investigations on a behavioral level (Granacher et al., 2010; Keller et al., 2012; Donath et al., 2013, 2016; Pfusterschmied et al., 2013), slacklining training is known for its specific and task-related effects (Donath et al., 2017; Serrien et al., 2017; Giboin et al., 2018) and is considered complementary to conventional sensorimotor training (Volery et al., 2017). However, in contrast to the above-mentioned balance tasks and instruments with limited degrees of freedom, evidence on the underlying neuronal mechanisms of slacklining is rather sparse. Only a few recent studies using MRI measurements investigated neural adaptations subsequent to a slacklining intervention over several weeks. While two studies observed training-induced structural brain adaptations (Dordevic et al., 2018, 2020), two other studies revealed functional connectivity changes subsequent to slacklining training in brain regions associated with posture and balance control (Magon et al., 2016; Giboin et al., 2019). However, to date, there is a lack of knowledge with regards to capturing brain activity during the execution of slacklining tasks, although wearable non-invasive brain imaging techniques have proven their feasibility even in unconstrained sports-related environments (Seidel-Marzi and Ragert, 2020).

Hence, the aim of the present study was to assess hemodynamic response alterations by means of fNIRS during the execution of slacklining in advanced slackliners. In order to disentangle the influence of task difficulty on brain processing, we aimed at investigating differences between standing (ST) and walking (WA) on a slackline as two conditions. Differences on physiological and behavioral levels between both conditions were evaluated based on drops from the slackline, perceived level of task difficulty and changes in heart rate (HR). On a neuronal level, according to previous balance-related findings, we hypothesized that (a) sensorimotor brain regions involved in motor planning, preparation, and execution such as M1, premotor cortex (PMC), and supplementary motor cortex (SMA) are involved in both slacklining conditions (Herold et al., 2017b). Furthermore, we expected to observe (b) task-related lower hemodynamic responses during ST as compared to WA (Verstynen et al., 2005; Holper et al., 2009). Additionally, in accordance with the “neural efficiency” hypothesis (Dunst et al., 2014), we hypothesized that (c) slacklining experience (in years) is associated with lower levels of cortical activation during both conditions. This assumption is based on previous studies revealing lower brain activity in trained athletes as compared to lower-level athletes/non-athletes while performing the same task (Naito and Hirose, 2014; Seidel et al., 2017).

## Materials and Methods

### Ethical Approval

The study was approved by the local ethics committee of the Medical Faculty at the University of Leipzig (309/17-ek). All participants gave written informed consent to participate in the experiments according to the Declaration of Helsinki.

### Participants

A total number of 16 healthy participants [mean age: 27.69 ± 0.86 years (mean ± SE); range 20–32 years; three females] were enrolled in the present study. None of the participants had a history of neurological illness, and during the time of the experiment, none of the volunteers was taking any central-acting drugs. All participants were right-handed according to the Edinburgh Handedness Inventory (mean handedness score: 79.41 ± 5.25; Oldfield, 1971). Total hours of sports per week (mean: 7.09 ± 1.87 h), hours of fine-motor training/activity per week (e.g., playing a musical instrument, knitting, doing handcrafts, playing video games, mean: 1.94 ± 0.73 h) as well as the extent of slacklining activities in the past and present were assessed prior to testing. Hence, participants were recruited based on their slacklining experience (mean: 7.25 ± 0.68 years) and their current slacklining activities (mean: 4.09 ± 0.56 h per week) and were therefore considered as advanced slackliners.

### Experimental Procedure

**Figure 1:**
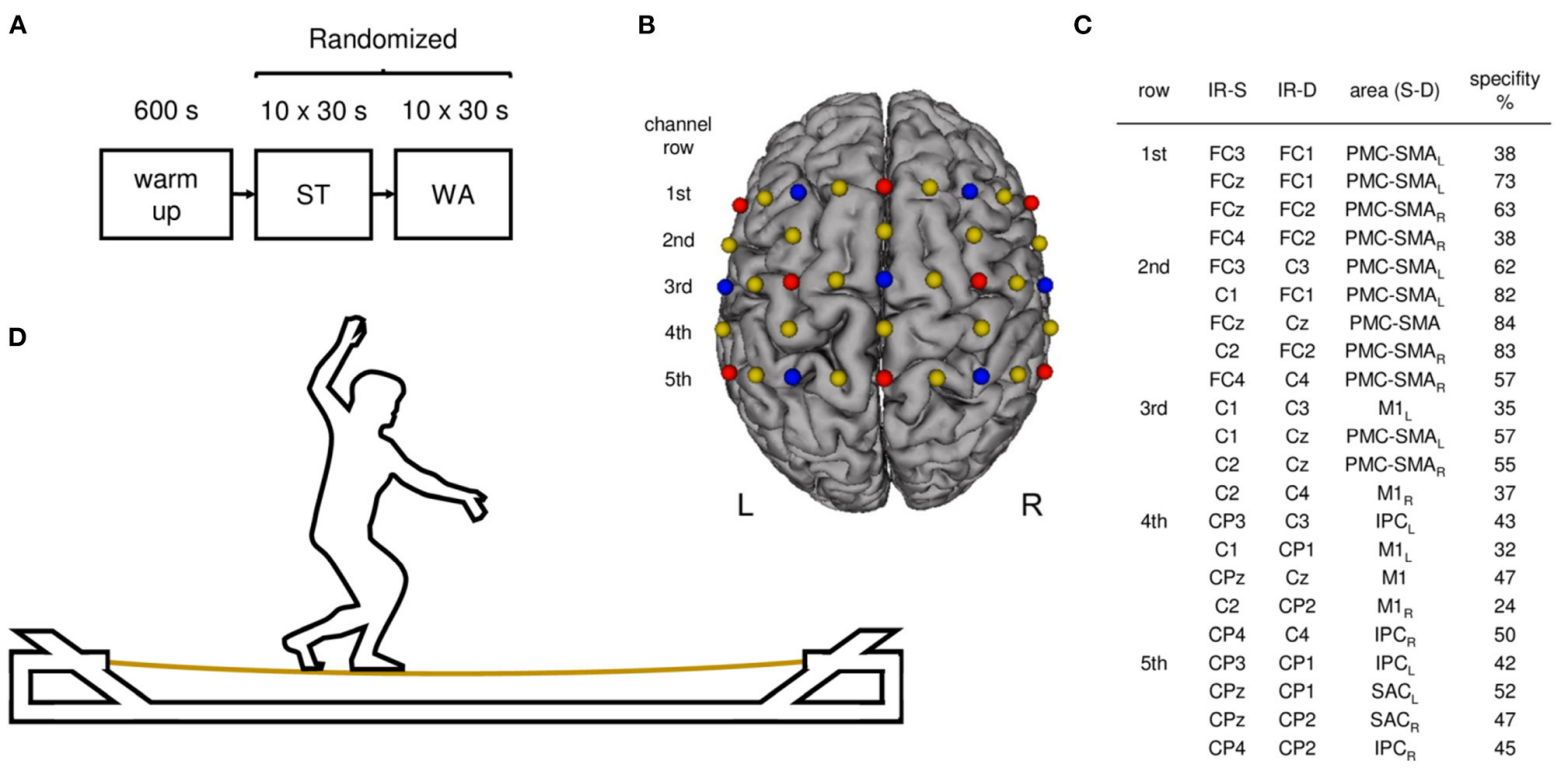
Study design and experimental setup. **(A)** Experimental procedure: participants started with a 10 min warm-up phase. Afterwards participants performed standing (ST) and walking (WA) on a slackline in randomized order. **(B)** Illustration of fNIRS configuration used during ST and WA. Sources are shown as red dots and detectors as blue dots. Yellow dots represent each center of the 22 channels (inter-optode distance 3 cm). **(C)** 10–20 positions for infrared sources (IR-S) and detectors (IR-D), respective brain regions (arranged in rows), targeted by a 10–20 system transfer method and defined by the “Brodman” Atlas (PMC-SMA, premotor and supplementary motor cortex; SAC, somatosensory association cortex; M1, primary motor cortex; IPC, inferior parietal cortex; L, left hemisphere, R, right hemisphere; Zimeo Morais et al., 2018). **(D)** Participant during task execution on a solid slacklining frame.

The present study on advanced slackliners aimed at quantifying hemodynamic response alterations during standing on a slackline (ST) and walking on a slackline (WA) using a solid slacklining frame (Slackstar, Braun GmbH, Neurmarkt, Germany). The slackline had a length of 4 m and a height of 50 cm with small platforms at each end at the same height (see Figure 1D). Using a within-subject design, both conditions were performed in a block-wise manner for 10 × 30 s (30 s resting phases between trials) in one session (see Figure 1A). The order of conditions was randomized; however, all trials of each condition were performed consecutively in one block.

During WA, participants were asked to walk forward over the slackline at a preferred pace. Platforms were used for direction changes and inter-trial resting phases, respectively. During ST, participants were asked to stand in the middle of the slackline without changing their standing leg. In case of a loss of balance (drops from the slackline), participants were asked to get back on the slackline immediately. Motor performance was videotaped and assessed as the number of drops per trial. Additionally, after each condition participants were asked to rate the perceived level of task difficulty on a scale from 1 (very easy) to 10 (very difficult). Moreover, a chest strap (Polar Electro Oy, Kempele, Finland) was applied to assess HR. Before and after the entire experiment, participants rated their levels of attention, fatigue, and discomfort on a visual analog scale (VAS).

### Functional Near-Infrared Spectroscopy (fNIRS)

Hemodynamic responses during ST and WA were recorded using the portable NIRSport system (NIRx Medical Technologies, Glen Head, NY) covering sensorimotor brain areas [premotor and supplementary motor cortex (PMC–SMA), primary motor cortex (M1), somatosensory association cortex (SAC), and inferior parietal cortex (IPC)] on both hemispheres (see Figure 1C). Using a total of eight light sources and seven detectors with an inter-optode distance of 30 mm, hemodynamic responses were measured in 22 channels (see Figure 1B). Optodes were placed on an fNIRS cap (with different sizes according to participants' head sizes) including optode distance holders, which ensured fixed source-detector distances and standardized sensor placement according to the international 10–20 system. Infrared light was emitted by sources with wavelengths of 760 and 850 nm. The NIRSport system uses time and frequency multiplexing to minimize crosstalk between wavelengths and optodes and acquires data with a sampling frequency of 7.81 Hz.

### Data Analysis

#### Hemodynamics

fNIRS data analysis was performed using the MATLAB-based (MathWorks, Natick, MA, United States of America, R2020a) HOMER2 toolbox (Huppert et al., 2009). Statistical analysis was conducted using RStudio 1.1.383 (RStudio Team, 2020).

Raw fNIRS recordings were pre-processed in order to reduce the influence of motion artifacts and physiological noise (according to Carius et al., 2020a) before estimating concentration changes of oxygenated and deoxygenated hemoglobin (ΔHbO_2_ and ΔHHb, respectively). According to this procedure, only 3.7% (ST) resp. 2.8% (WA) of the channels were regarded as too noisy and therefore not included in further analysis steps. In a first step, raw intensity signals were converted to changes in optical density (Huppert et al., 2009). We used principal component analysis (PCA) in order to separate the neurovascular component from the systemic noise (Yücel et al., 2021). We filtered out the first principal component (HOMER2 *enPCAFilter* function; nSV = 1), which has the highest correlation with the global average signal (Hocke et al., 2018). Correction for motion artifacts was performed using a hybrid method that takes advantage of different correction algorithms, so-called Spline interpolation with Savitzky-Golay (SG) filtering (Jahani et al., 2018). We used the algorithm described by Jahani et al. (2018) as implemented in the HOMER2 *hmrMotionCorrectSplineSG* filtering function (*p* = *0.99, FrameSize_sec* = *6*, Jahani et al., 2018). Subsequently, data were band-pass filtered (HOMER2 *hmrBandpassFilt* function; 3rd-order Butterworth bandpass filter) to attenuate low frequency drift, Mayer wave, breathing rate, and HR components using 0.01 Hz as high and 0.09 Hz as low pass cutoff frequencies (Pinti et al., 2018).

In a further step, attenuation changes of both wavelengths (850 and 760 nm) were transformed to concentration changes (Δ) of HbO_2_ and HHb using the modified Beer–Lambert approach (partial pathlength factor: 6.0; Huppert et al., 2009). Although ΔHHb is considered as the more valid parameter to evaluate alterations in hemodynamic response since it is less contaminated by extracerebral processes (Kirilina et al., 2012), reporting ΔHbO_2_ and ΔHHb (instead of only one of both) is strongly recommended in current literature and allows better physiological interpretation of the functional experimental results (Tachtsidis and Scholkmann, 2016).

Single trials were baseline corrected (regarding 5 s until stimulus onset) and time courses of ΔHbO_2_ and ΔHHb in each channel were block-averaged using the HOMER2 *hmrBlockAvg* function. Though the experimental block design included resting phases to prevent overlapping of the hemodynamic responses between trials, we directly analyzed the height of amplitude (baseline-corrected average of the temporal window from 5 to 30 s with regard to stimulus onset for slacklining condition; Herold et al., 2017b).

In order to evaluate contrasts between task-related hemodynamic response alterations during slacklining conditions and baseline, dependent *t*-tests were conducted in a channel-wise manner. We applied *robust statistical tests* (Wilcox, 2017), since the assumptions for parametric tests (e.g., normal distribution) are often violated for fNIRS data (Santosa et al., 2018). Differences between ST and WA were tested using robust two-sample trimmed mean *t*-tests. These robust statistical tests (function *yuend*, trim = 0.2) were conducted in R using the *WRS2* software package (Mair and Wilcox, 2017). As suggested by Wilcox and Tian (2011), an *explanatory measure of effect size* ξ was reported, while values of ξ = 0.10, 0.30, and 0.50 correspond to small, medium, and large effect sizes. To control for multiple comparisons during robust *t*-tests we applied the false discovery rate (FDR) correction (*q* < 0.05; Singh and Dan, 2006). The resulting channel-wise *t*-values were mapped using Brain Function Mapping Tool from Wang et al. (2016). Additionally, a robust percentage bend correlation analysis (Mair and Wilcox, 2017; Wilcox, 2017) was performed in order to determine the relationship between task-related hemodynamic response alterations and slacklining experience (in years). For all tests, a *p*-value of < 0.05 was considered significant.

#### Behavior

On a behavioral level, drops from the slackline were counted as a parameter to assess slacklining performance. Drops were averaged for both conditions (ST vs. WA) and statistically analyzed using SPSS Statistics 22 (IBM, Armonk, NY). Since Shapiro–Wilk test revealed that data were not normally distributed, differences between conditions were analyzed using Wilcoxon test. The same procedure was applied to the ratings of perceived level of task difficulty. For all tests, a *p*-value of < 0.05 was considered significant.

#### Heart Rate

Heart rate (HR) was assessed for each trial during both slacklining conditions and averaged across all participants. Statistical analysis was conducted using SPSS Statistics 22 (IBM, Armonk, NY). Shapiro–Wilk test revealed that HR data were normally distributed. Therefore, a 10 × 2 repeated measures (rm)ANOVA was performed to compare HR alterations over 10 trials (first within-subject factor) during both conditions (ST vs. WA as second within-subject factor). Additionally, *post-hoc* tests (dependent *t*-tests) were used to analyze the differences if necessary. A *p*-value of < 0.05 was considered significant.

## Results

### Behavioral Data

**Figure 2:**
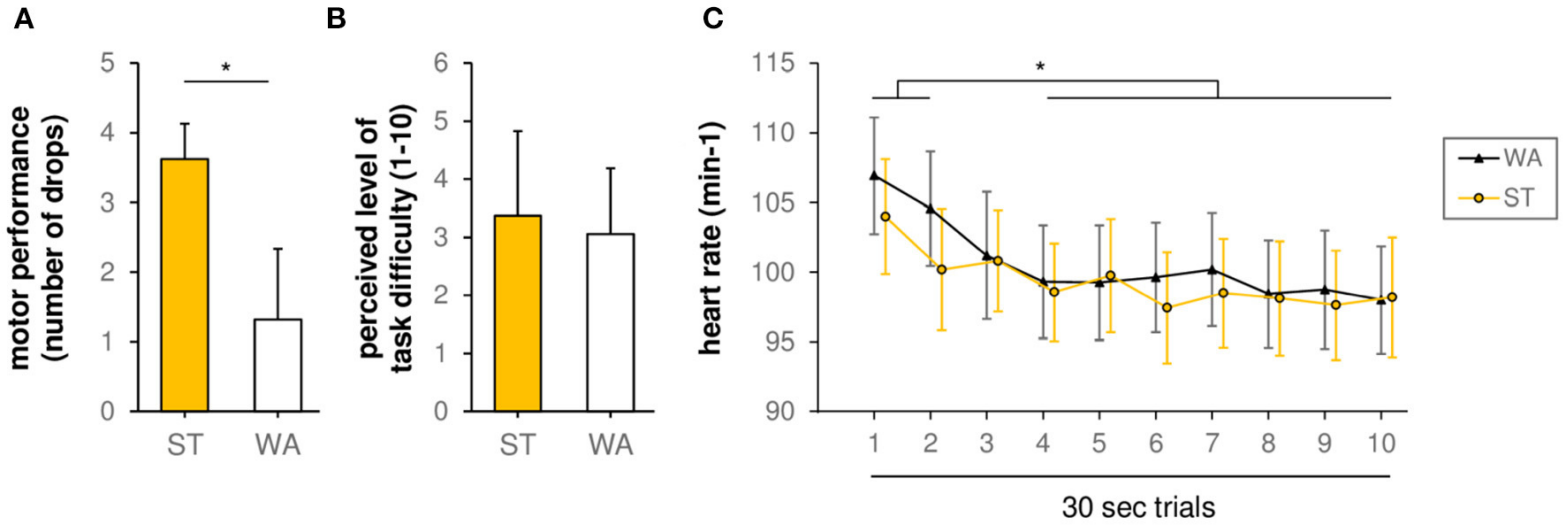
**(A)** Motor performance during standing (ST) and walking (WA) on a slackline (grand-average results, *n* = 16, number of drops). *(*p* < 0.05) indicates significant differences between conditions. **(B)** Perceived level of task difficulty during ST and WA (1 = very easy, 10 = very hard). **(C)** Heart rate during ST and WA. *(*p* < 0.05) indicates significant differences between trials.

The comparison of slacklining conditions on a behavioral level revealed a significant difference (*p* = 0.019, Z = −2.354) between WA [1.31 ± 0.51 (mean ± SE) drops] and ST (3.63 ± 1.02 drops) (see Figure 2A). However, with regards to perceived level of task difficulty, Wilcoxon test revealed no differences (*p* = 0.317, Z = −1.000, see Figure 2B) between WA (3.06 ± 0.28) and ST (3.38 ± 0.36).

### Heart Rate

With regards to HR, rmANOVA revealed a non-significant trial × condition interaction [*F*_(1, 9)_ = 1.220, *p* = 0.289, η^2^_*p*_ = 0.080]. Moreover, we found no significant influence of factor condition on HR [main effect of factor condition: *F*_(1, 14)_ = 1.008, *p* = 0.332, η^2^_*p*_ = 0.067]. Only factor trial showed a significant influence on HR [main effect of factor trial: *F*_(9, 126)_ = 170.917, *p* = 0.001, η^2^_*p*_ = 0.413], indicating alteration in HR over time during both conditions (see Figure 2C). In detail, *post-hoc* tests revealed a significant decline in HR between trial 1 and trial 3–10 as well as trial 2 and trial 4–10 [*t*_(15)_ = 3.53–5.39, *p*_adjusted_ < 0.024].

### Hemodynamics

#### Hemodynamic Response Alterations During Standing on a Slackline

**Figure 3:**
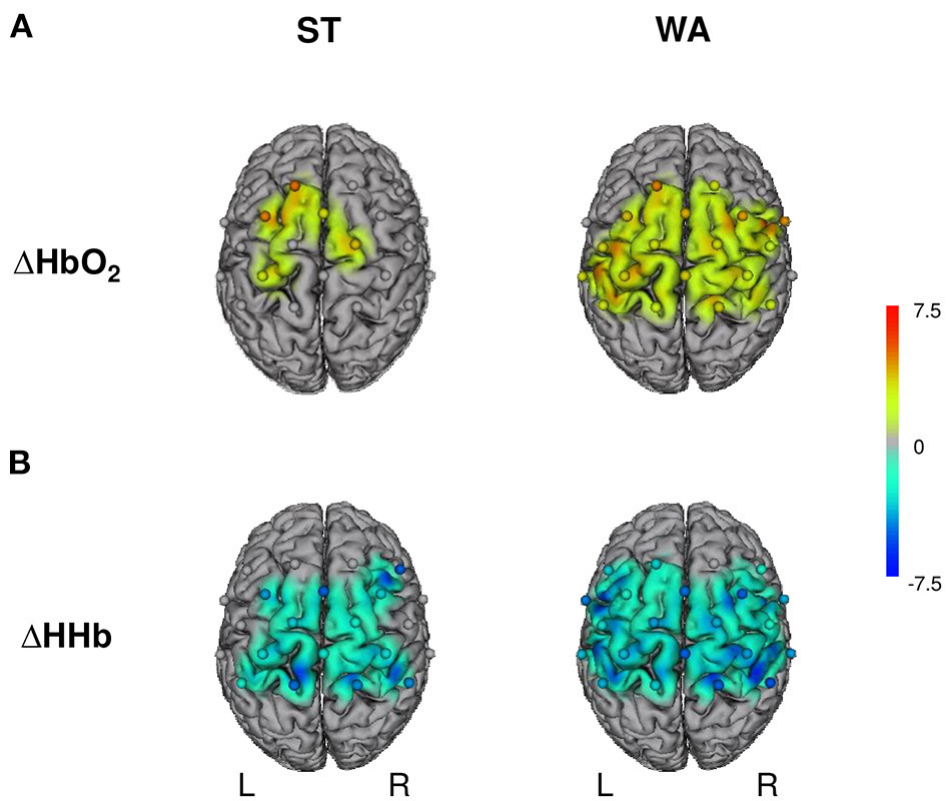
Hemodynamic responses during standing (ST) and walking (WA) on a slackline. L, left hemisphere; R, right hemisphere; ΔHbO_2_/ΔHHb, oxygenated/deoxygenated hemoglobin concentration changes. **(A)** Illustrates results for changes in ΔHbO_2_ and **(B)** for changes in ΔHHb; *n* = 16, *t*-maps, robust dependent sample mean tests (Mair and Wilcox, 2017; Wilcox, 2017). Activation maps with cortically projected channel positions (dots centered between source-detector pairs). Only *t*-values accompanied with FDR corrected *p*-values are shown.

Grand-averages of ΔHbO_2_ and ΔHHb indicate that ST induced task-related hemodynamic response alterations in sensorimotor brain areas (negative correlation between ΔHbO_2_ and ΔHHb). With regards to ΔHbO_2_ concentrations, comparisons between ST and baseline revealed significant increases in FCz–Cz (PMC–SMA), C1–FC1, FCz–FC1 (PMC–SMA_L_), C2–Cz (PMC–SMA_R_), and C1–CP1 (M1_L_) only (3.19 ≤ *t* ≤ 5.00, 0.001 ≤ *p* ≤ 0.011, FDR adjusted *q*-value of 0.011, see Figure 3A). In contrast, significant decreases of ΔHHb concentrations were found in most channels (−5.08 ≤ *t* ≤ −3.73, 0.001 ≤ *p* ≤ 0.028, FDR adjusted *q*-value of 0.030, see Figure 3B), with the exception of C1–C3 (M1_L_), C2–C4 (M1_R_), FC3–C3, FC3–FC1, FCz–FC1 (PMC–SMA_L_), FCz–FC2, FC4–C4 (PMC–SMA_R_), CP3–C3 (IPC_L_), and CP4–C4 (IPC_R_). Typically, the effects are on average greater in ΔHbO_2_ than in ΔHHb.

#### Hemodynamic Response Alterations During Walking on a Slackline

Typical task-related hemodynamic response alterations in sensorimotor brain areas were also found during WA. ΔHbO_2_ concentrations increased significantly in all channels (2.54 ≤ *t* ≤ 4.73, 0.001 ≤ *p* ≤ 0.031, FDR adjusted *q*-value of 0.41, see Figure 3A), with the exception of FC3–C3, FC3–FC1 (PMC–SMA_L_), FC4–FC2 (PMC–SMA_R_), and CP4–C4 (IPC_R_). Significant decreases of ΔHHb concentrations were found in all channels (−5.25 ≤ *t* ≤ −2.41, 0.001 ≤ *p* ≤ 0.039, FDR adjusted *p*-value of 0.048, see Figure 3B), except one (FCz–FC2: PMC–SMA_R_).

#### Comparison Between Standing and Walking on a Slackline

The comparison of task-related hemodynamic response alterations during ST and WA revealed no significant differences between conditions, neither for ΔHbO_2_ (−2.34 ≤ *t* ≤ 0.84, 0.044 ≤ *p* ≤ 0.989, FDR adjusted *q*-value of 0.002) nor for ΔHHb (−0.90 ≤ *t* ≤ 2.34, 0.044 ≤ *p* ≤ 0.941, FDR adjusted *q*-value of 0.002).

#### Effects of Slacklining Experience on Hemodynamic Response Alterations

Slacklining experience (in years) does not correlate with HR during ST [*r*_(16)_ = −0.24, *p* = 0.37] or WA [*r*_(16)_ = −0.31, *p* = 0.24]. Therefore, we conclude that cardiac parameters did not mediate the relationship of slacklining experience and hemodynamic response. Correlation analyses for task-related hemodynamic response alterations revealed that slacklining experience does not correlate with ΔHbO_2_ (WA: −0.33 ≤ *r* ≤ 0.37, 0.156 ≤ *p* ≤ 0.901; ST: −0.56 ≤ *r* ≤ 0.42, 0.045 ≤ *p* ≤ 0.994). We also found no significant association between slacklining experience and ΔHHb (WA: −0.35 ≤ *r* ≤ 0.22, 0.200 ≤ *p* ≤ 0.986; ST: −0.27 ≤ *r* ≤ 0.60, 0.014 ≤ *p* ≤ 0.985). Without FDR correction, data revealed a negative correlation between slacklining experience and ΔHbO_2_ in FCz–FC2 (PMC–SMA_R_, *r* = −0.56, *p* = 0.045) as well as a positive correlation between slacklining experience and ΔHHb in C2–C4 (M1_R_, *r* = 0.60, *p* = 0.014) during ST, both indicating that more slacklining experience might be associated with lower hemodynamic response alterations.

## Discussion

In the present study, we investigated hemodynamic response alterations during the execution of slacklining as a challenging whole-body balance task. By examining advanced slackliners, we also contributed to a relatively unexplored field, as brain functioning during expert task execution was only targeted in a small number of studies so far. In our study, participants executed standing and walking on a slackline as two different conditions in order to reveal task-related neural correlates of slacklining.

### Hemodynamic Response Alterations During Standing and Walking on a Slackline

We hypothesized to observe an involvement of sensorimotor brain areas such as M1, PMC, and SMA, since both standing and walking on a slackline are considered demanding and represent multisensory balance tasks (hypothesis a). Indeed, we found hemodynamic response alterations in the above-mentioned regions for both chromophores during both slacklining conditions. These results go in line with previous studies investigating the crucial role of sensorimotor brain areas for balance control (Hiyamizu et al., 2014; Herold et al., 2017a; Seidel et al., 2017). For example, similar to our findings, Herold et al. (2017a) recently observed enhanced cortical activity in SMA, precentral gyrus (PrG), and postcentral gyrus (PoG) using fNIRS during the execution of a balance task on a balance board. Similar activation pattern were found in another recent study by Seidel et al. (2017) in endurance athletes and non-athletes. Moreover, Herold et al. (2017a) even found a strong negative correlation between the magnitude of SMA activity and sway in mediolateral direction during task execution using an additional inertial sensor. In another fNIRS study, ΔHbO_2_ concentrations in SMA were increased after a single training session on a balance board (Hiyamizu et al., 2014). These findings suggest a crucial involvement of sensorimotor brain areas such as M1, PMC, and SMA in balance control since different sensory information are integrated and processed in these brain regions during the execution of a motor task (Nachev et al., 2008).

Based on the above mentioned results, hemodynamic response alterations in sensorimotor brain areas during balancing on a balance board and also during slacklining can be explained by indirect and direct locomotor pathways as proposed by Herold et al. (2017b). According to the authors, PMC and SMA are part of an indirect locomotor pathway indicating a more controlled task execution. More automatic motor control, in contrast, is realized via a direct locomotor pathway, which includes M1 and other brain regions such as the cerebellum. The fact that we observed an increased brain activity in areas of both the direct and the indirect locomotor pathway indicates that slacklining, even by advanced slackliners, can neither be exclusively assigned to a controlled nor an automatic motor control during task execution. In fact, the demands of slacklining seem to be more complex (higher number of degrees of freedom) that both controlled and automatic components of motor control are required for task execution. Therefore, it remains speculative whether experienced slackliners would show less and/or more focal cortical activity during balancing on a balance board as compared to balance untrained participants, which should be addressed more precisely in future studies.

### No Task-Related Hemodynamic Response Alterations During Slacklining Conditions

We further hypothesized to detect task-related differences in hemodynamic response alterations between slacklining conditions (hypothesis b). Indeed, we observed no differential neural effects between ST and WA condition, which might be explained on a behavioral level by the fact that participants did not perceive any differences regarding the task complexity of both conditions. However, the significant differences in motor performance (drops from slackline) in turn point to a differential task complexity, interestingly toward a more challenging ST condition. This in turn might be explained by the fact that ST might be rather untypical for slacklining, requires altered motor control as compared to WA and therefore causes a higher number of drops. On the one hand, the lack of task-related differences in brain processing may be attributed to methodological issues discussed in the limitation section.

On the other hand, to date, investigations of task-related neuronal effects using fNIRS are rather sparse. However, as previously demonstrated by Holper et al. (2009), fNIRS is indeed capable of detecting differences in hemodynamic response alterations as a function of task complexity. In this study, participants performed five different finger tapping tasks with increasing task complexity: unimanual simple and complex tasks (each with left and right hand) as well as bimanual complex tasks. The authors reported significant hemodynamic response alterations in M1 for both ΔHbO_2_ and ΔHHb between all conditions (Holper et al., 2009), confirming evidence on hand/finger tapping tasks from previous fMRI studies (Solodkin et al., 2001; Verstynen et al., 2005; Witt et al., 2008). Based on this evidence, it can therefore be assumed that it is crucial for observing differential task-related effects whether the task is executed unilaterally or bilaterally. This is further supported by a recent fNIRS investigation by Carius et al. (2020b), where participants performed a complex basketball slalom dribbling task. Here, the authors found that task-related hemodynamic response alterations in M1 are related not only to laterality but also to the pace of task execution. Hence, the lack of differential hemodynamic response alterations between conditions in our results might be attributed to the fact that both ST and WA were performed bipedally and also the upper extremities each realized comparable support functions. Further explanations, however, remain purely speculative since, to our knowledge, there is no further evidence to date regarding differential task-related neuronal effects in lower limb or whole-body motor tasks.

### Influence of Experience on Hemodynamic Response During Slacklining Conditions

In addition, we performed a correlation analysis investigating the effect of slacklining experience on hemodynamic response alterations during slacklining (hypothesis c). Here, we found no significant correlation for both chromophores, neither for ST nor for WA. However, without applying FDR-correction, our results revealed that slacklining experience might be associated with lower hemodynamic response alterations during ST. This finding can be considered a tentative indication that more experienced slackliners exhibit less cortical activity, mainly within right PMC–SMA and right M1. This trend would be consistent with previous studies showing that experts have more efficient neural networks while performing motor tasks (Seidel et al., 2017; Carius et al., 2020a) as proposed by the “neural efficiency” hypothesis (Dunst et al., 2014). Moreover, less cortical activity in PMC–SMA might indicate a greater automaticity and fine tuning of the motor performance in higher-skilled individuals, as automaticity in the motor learning process is associated with a decrease in frontal and secondary motor areas (Poldrack et al., 2005). This would also be the case for less cortical activity in M1, which, as part of a direct locomotor pathway, also points to automaticity of a motor/balance task (Herold et al., 2017b). All in all, however, the uncorrected findings of the correlation analysis need to be considered with caution and the potential associations need to be retested using a larger sample size with a bigger range of expertise including also novices and professionals.

From a methodological point of view, another explanation for the absence of significant associations between slacklining experience and hemodynamic response alterations might be attributed to the method used for rating the participants, since only slacklining experience in years was surveyed. More slacklining experience, however, is not necessarily associated with a higher slacklining expertise and/or superior slacklining performance. Hence, future studies on experts should find a solution to operationalize the slacklining skills more precisely. In addition, further behavioral parameters (e.g., body sway, pace of execution, number of steps, vibration of the slackline, etc.) should be recorded during task execution in order to quantify slacklining performance and to explain related hemodynamic response alterations more profoundly. Indeed, this would also be conceivable by using state-of-the-art approaches such as motion capturing, allowing the quantification of body part movements (e.g., arms) and the identification of potential (balance) execution strategies.

### Study Limitations

In the present study, hemodynamic response alterations were assessed during slacklining, which can be considered a challenging whole-body balance task. Therefore, only advanced slackliners were recruited, who were able to perform both conditions of the task reliably in a block design consisting of multiple trials. Since slacklining novices were expected not to be able to perform this experimental procedure reliably without causing a minor data quality, an additional novice control group was not included. This, however, came with the cost of a missing classification of the results as specific for advanced slackliners. From a methodological perspective it is worth mentioning that hemodynamic response alterations were only captured in sensorimotor brain areas since this was the main focus of the present study. There is no doubt, however, that other brain areas outside the human motor system are also involved in the execution of a whole-body balance tasks such as slacklining. Hence, future studies should address this issue using whole-brain fNIRS montages. Furthermore, we only investigated hemodynamic response alterations within cortical sensorimotor brain areas and not in extra- and/or sub-cortical regions, which is due to the limited penetration depth of fNIRS. Beyond that, it is necessary to mention that whole-body balance tasks such as slacklining might lead to motion artifacts, e.g., caused by head movements, influencing the fNIRS signal. Therefore, we applied state-of-the-art motion artifact and baseline correction methods to minimize the effect of artifacts on the data. In future studies, further influences on the fNIRS signal such as physiological confounders (e.g., scalp blood flow) should be reduced by using multi-distance fNIRS measurements. Furthermore, additional physiological data assessments such as respiratory measurements might help quantifying the physical effort of the slacklining task more precisely and monitoring systematic influences.

## Conclusion

Taken together, the present proof-of-concept study conducted for the first time measurements of hemodynamic response alterations during slacklining as a challenging whole-body balance task. We quantified neural correlates in sensorimotor brain regions such as M1, PMC, and SMA during standing and walking on a slackline in advanced slackliners without observing differential effects between conditions or associations with slacklining experience. On the one hand, these findings contribute to a relatively unexplored field, as brain functioning during expert task execution was only targeted in a small number of studies so far. On the other hand, our results provide novel insights by extending established balance tasks contributing to a more profound understanding of neural correlates of whole-body balance tasks. This, in turn, has implications beyond sports and expert research, as maintaining balance is considered an important requirement in daily life and crucial in the context of prevention and rehabilitation.

## Data Availability Statement

The raw data supporting the conclusions of this article will be made available by the authors, without undue reservation.

## Ethics Statement

The studies involving human participants were reviewed and approved by Ethics committee of the Medical Faculty at the University of Leipzig. The patients/participants provided their written informed consent to participate in this study.

## Author Contributions

All experiments were conducted at the Faculty of Sport Science of the University of Leipzig. PR, OS-M, DC, and SH designed the study and experimental set-up. Participants were recruited and tested by SH. DC analyzed data and created figures in cooperation with OS-M. DC and OS-M prepared the manuscript and revised/finalized it in cooperation with PR. All authors interpreted data, contributed to the manuscript, reviewed it, approved the content of the final version, and agree to be accountable for all aspects of the work. All persons designated as authors qualify for authorship, and all those who qualified for authorship are listed.

## Conflict of Interest

The authors declare that the research was conducted in the absence of any commercial or financial relationships that could be construed as a potential conflict of interest.
